# Fatty Liver and Insulin Resistance in the Liver-Specific Knockout Mice of Mitogen Inducible Gene-6

**DOI:** 10.1155/2016/1632061

**Published:** 2016-12-07

**Authors:** Byung Kil Park, Eun-Ah Lee, Hee-Youn Kim, Jun Choul Lee, Koon Soon Kim, Won Hoon Jeong, Ki Young Kim, Bon Jeong Ku, Sang Dal Rhee

**Affiliations:** ^1^Research Center for Drug Discovery Technology, Division of Drug Discovery Research, Korea Research Institute of Chemical Technology, Daejeon, Republic of Korea; ^2^Department of Drug Development and Discovery, Graduate School of New Drug Development and Discovery, Chungnam National University, Daejeon, Republic of Korea; ^3^Department of Internal Medicine, Chungnam National University School of Medicine, Daejeon, Republic of Korea

## Abstract

Mitogen inducible gene-6 (Mig-6) is a feedback inhibitor of epidermal growth factor receptor (EGFR) signaling pathway. The liver-specific knockout mice of the Mig-6 gene (Mig-6^*d/d*^) showed hepatomegaly and increased hypercholesterolemia. In this study, the biomarkers of insulin resistance and the effects of high-fat diets in the wild (Mig-6^*f/f*^) and Mig-6^*d/d*^ mice were analyzed. The fasting plasma concentrations of glucose, triglyceride, cholesterols, free fatty acids, and HOMA-IR were measured and the glucose tolerance and insulin resistance tests were performed in the 25-week-old Mig-6^*f/f*^ and the Mig-6^*d/d*^ mice. The protein levels of active insulin receptor, glucose 6-phosphatase, and phosphoenolpyruvate carboxykinase were analyzed in the liver and fat. The fasting plasma cholesterol and glucose concentration were higher in the Mig-6^*d/d*^ mice than the Mig-6^*f/f*^ mice with increased fat deposition in the liver. But the Mig-6^*d/d*^ mice had the improved glucose intolerance and insulin resistance without increased amount of phosphoinsulin receptor after insulin infusion in the liver. The hepatic concentration of phosphoenolpyruvate carboxykinase was increased in fasting Mig-6^*d/d*^ mice. The feeding of high-fat diet accelerated the plasma lipids profiles and HOMA-IR in the Mig-6^*d/d*^ mice but had no differential effects in oral glucose tolerance test and insulin tolerance test in both genotypes. These results suggest that the activated EGFR signaling might increase the fasting plasma glucose concentration through inducing the hepatic steatosis and the improved whole-body insulin resistance in the KO mice be caused by decreased adipogenesis in fat tissues.

## 1. Introduction

Mitogen inducible gene-6 (Mig-6) is an adaptor protein that is highly expressed in the liver and kidney [1]. Its expression is induced by various growth factors, stresses, and hormones, such as epidermal growth factor (EGF), tumor growth factor-*α* (TGF-*α*), insulin, insulin like growth factor-1 (IGF-1), hypoxia, and glucocorticoid [2–5]. The Mig-6 protein contains several domains which are important in interacting with various signaling molecules such as Cdc42/Rac-interaction binding (CRIB) domain and a Src-homology (SH3) domain [6].

The major function of Mig-6 is a negative feedback regulator of epidermal growth factor receptor (EGFR) signaling pathway [7]. The decreased expression of Mig-6 was observed in human breast cancer, which is correlated with reduced overall survival of breast cancer patients [8], and loss of the protein's function contributes to initiation of several cancers such as lung cancer [5]. Therefore, it is known that Mig-6 is a tumor-suppressor gene. The EGFR signaling pathway is activated by binding of ligands, EGF, or TGF-*α* which is closely associated with cancer [9]. The EGFR is overexpressed in most human cancers, including breast cancer [10], cervical cancer [11], and lung cancer [12]. An EGFR specific inhibitor, gefitinib, is successfully used for treating several cancers [13].

When some EGFR inhibitors were given to the patients suffering from both cancer and diabetes mellitus, it not only treated cancer but also improved diabetes mellitus [14]. Furthermore, treatment of an EGFR inhibitor (PD153035) improves glucose intolerance and insulin sensitivity and reduces inflammation in diet-induced obese (DIO) mice [15], indicating the some roles of the EGFR signaling pathway in the pathophysiologies of diabetes mellitus. Additionally, liver-specific knockout mice of Mig-6 gene revealed hypercholesterolemia and fatty liver [16]. This report suggested that activation of EGFR signaling pathway in the liver might disrupt whole-body insulin resistance. So, we analyzed the diabetes mellitus-related biomarkers of liver-specific knockout mice of Mig-6 (Mig-6^*d/d*^) and the effects of high-fat diet on the phenotypes.

## 2. Materials and Methods

### 2.1. Animals and Treatments

Mig-6^*d/d*^ and control mice (Mig-6^*f/f*^) were maintained in the animal facilities of Korea Research Institute of Chemical Technology (KRICT) and used for experiments according to* the Guidelines for Animal Experimentation* under admission of* the Institutional Animal Care and Use Committee (IACUC)* of KRICT. All animals were maintained in a room illuminated daily from 07:00 to 19:00 (12:12 h light/dark cycle), temperature (23 ± 1°C), ventilation (10–12 times per hour), and humidity (55 ± 5%). Mice were caged individually and allowed free access to tap water and feed.

Mice were fed with a high-fat diet (HFD; Research Diet, D12492) or normal chow (NC) for 20 weeks from 5 weeks of age. Bodyweight was weekly monitored and food intake rates were biweekly measured. Some organs were weighted after sacrifice at the end of the experiment.

### 2.2. Analyses of Plasma Concentration Biochemical Parameters and Insulin

The blood samples were collected from overnight fasted mice. The concentrations of glucose, total cholesterol, high-density lipoprotein- (HDL-) cholesterol, low-density lipoprotein- (LDL-) cholesterol, triglyceride (TG), and nonesterified fatty acid (NEFA) in plasma were measured with colorimetric method using automated biochemical analyzer, Respons 920 (DiaSys, Germany). The plasma insulin levels were measured using the ultrasensitive mouse insulin ELISA kit (Shibayagi, Japan) according to the manufacturer's instructions. The HOMA-IR values, as an insulin sensitivity index, were calculated based on the following formula: (fasting insulin [uIU/mL] × fasting glucose [mg/dL])/405.

### 2.3. Oral Glucose Tolerance Test (OGTT) and Insulin Tolerance Test (ITT)

For OGTT, mice were orally given glucose at 2 g/kg bodyweight after overnight fasting. Blood samples were taken at time points of 0, 15, 30, 60, and 120 minutes from retroorbital plexus using heparin-coated capillary tubes. In ITT, insulin (Sigma, USA) was intraperitoneally injected with 0.54 IU/kg bodyweight and blood samples were taken at the same time point as OGTT. Rate constant for insulin tolerance test (KITT) was calculated using the formula K_ITT_ (%/min) = 0.693/*t*(1/2), where* t*(1/2) was calculated from the slope of plasma glucose concentration during 3–15 minutes after administration of intravenous insulin. The blood samples were immediately centrifuged at 1000 ×g for 10 min and the resulting plasma samples were stored at −20°C until being assayed.

### 2.4. Histological Analyses

After mice were sacrificed, the livers and white adipose tissues were collected, fixed in 10% formalin, embedded in paraffin, and sectioned at a thickness of 4~6 *μ*m. Hematoxylin and eosin (H&E) staining were performed. The immunostaining of paraffin-embedded sections of the reproductive fat tissues was performed using macrophage-specific antibody (Abcam, USA) and ABC staining kit (Santa Cruz, USA) for detecting the inflammation in the adipose tissues.

### 2.5. Western Blot Analysis

20 *μ*g of total protein was separated on NuPAGE 4–12% BIS-TRIS Gels (Invitrogen, USA) and transferred onto a PVDF membrane. Membrane was blocked in blocking buffer (Sigma, USA) for 1 hr. The immunoblotting was performed at 4°C overnight with shaking using antibodies against p-ERK (Cell Signaling, USA), p-IR, IR, PEPCK, G-6-Pase (Abcam, USA), ERK, p-PPAR*γ*, PPAR*γ*, and GAPDH (Santa Cruz, USA). After washing, the membrane was incubated in a 1 : 5000 dilution of a secondary antibody (goat anti-rabbit IgG, Thermo Scientific, USA) at room temperature for 1 hr. Protein bands were visualized using ECL kit (Thermo Scientific, USA).

### 2.6. Statistical Analysis

Statistical analysis was performed using Student's* t*-test. All data are reported as mean ± SEM. *∗*, *∗∗*, and *∗∗∗* indicate *P* < 0.05, *P* < 0.01, and *P* < 0.001, versus LFD group, respectively. #, ##, and ### indicate *P* < 0.05, *P* < 0.01, and *P* < 0.001 versus wild group, respectively.

## 3. Results

### 3.1. Food Intake Rates and Bodyweight Changes

**Figure 1:**
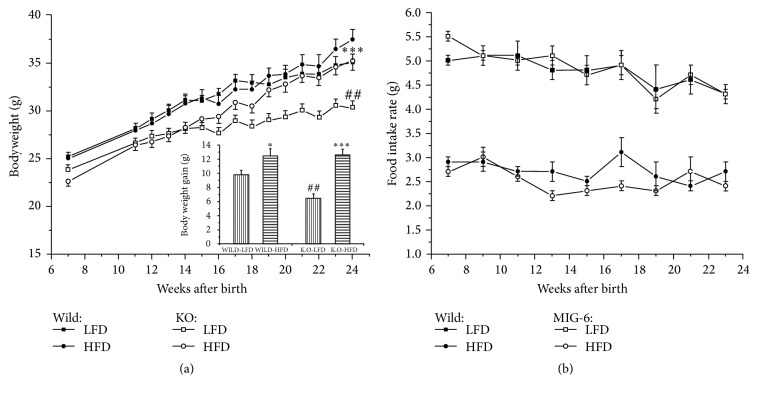
Bodyweight changes and effect of high-fat diet in Mig-6^*f/f*^ and Mig-6^*d/d*^ mice. (a) Bodyweight changes of Mig-6^*f/f*^ and Mig-6^*d/d*^ mice from 7 to 23 weeks of age. (b) Average food intake rate. *∗* and *∗∗∗* indicate *P* < 0.05 and *P* < 0.001, versus LFD treated group in the same genotype, respectively. ## indicate *P* < 0.01, versus Mig-6^*f/f*^. Data are presented as mean ± SE from 9 to 10 mice per group.

Effects of genotypes on food intake rates and bodyweights were measured. In NC group, the Mig-6^*d/d*^ mice displayed significantly lower bodyweight than the Mig-6^*f/f*^ mice through all ages. In HFD group, the Mig-6^*d/d*^ mice have lower bodyweights than Mig-6^*f/f*^ mice in almost all ages but not statistically significant at 25 weeks of age (Figure 1(a)). However, there were no significant differences between the genotypes in food intake rates (Figure 1(b)).

### 3.2. Plasma Concentrations of Biochemical Parameters

The plasma levels of lipids were analyzed after overnight fasting. In NC-fed groups, the Mig-6^*d/d*^ mice had elevated concentration of total cholesterol (*P* < 0.05) and HDL-cholesterol (*P* < 0.01) than Mig-6^*f/f*^ mice, which is the same as in the report of Ku et al. [16]. The HFD had no or little effects on the plasma concentration of total cholesterol, HDL-cholesterol, and LDL-cholesterol in Mig-6^*f/f*^ mice. In the Mig-6^*d/d*^ mice, however, HFD-fed mice had increased plasma concentration of total cholesterol (*P* < 0.001), HDL-cholesterol (*P* < 0.001), and LDL-cholesterol (*P* < 0.001) compared to NC-fed mice. The HFD also increased plasma TG levels in the Mig-6^*d/d*^ mice. These data suggested that the liver-specific deletion of Mig-6 might accelerate the effect of high-fat diet on plasma concentrations of cholesterols and TG (Table 1).

### 3.3. Glucose Tolerance Test, Insulin Resistance Tests, and Insulin Resistance Index

**Figure 2:**
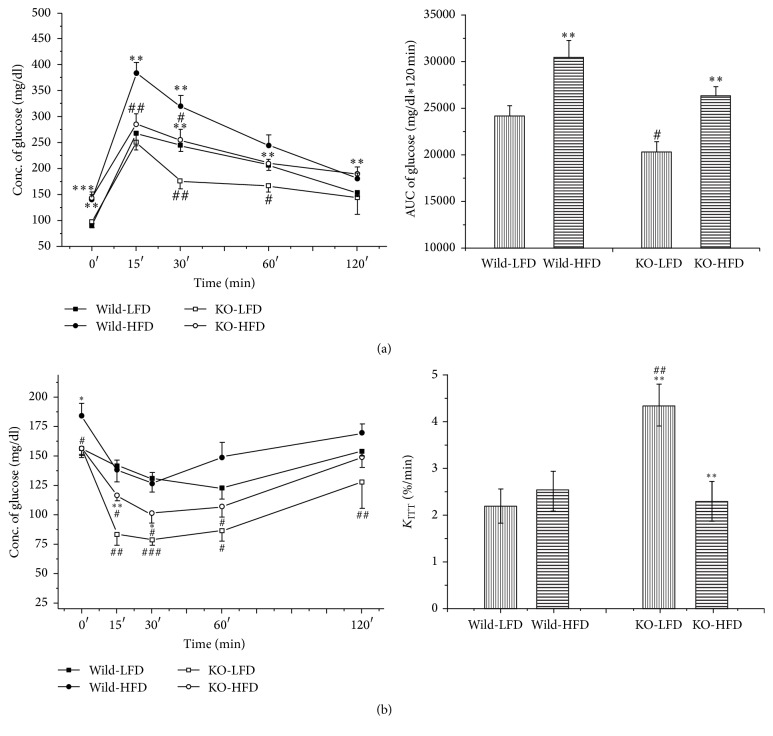
The oral glucose tolerance and insulin resistance test in Mig-6^*f/f*^ and Mig-6^*d/d*^ mice. (a) Oral glucose tolerance (left panel) and AUC of plasma plasma glucose concentration during the OGTT (right panel) at 23 weeks of age. (b) Change in plasma glucose concentration during insulin tolerance test (left panel), AUC of plasma plasma glucose concentration during the ITT (middle panel), and K_ITT_ (right panel) at 23 weeks of age. *∗*, *∗∗*, and *∗∗∗* indicate *P* < 0.05, *P* < 0.01, and *P* < 0.001, versus LFD treated group in the same genotype, respectively. #, ##, and ### indicate *P* < 0.05, *P* < 0.01, and *P* < 0.001, versus Mig-6^*f/f*^, respectively. Data are presented as mean ± SE from 9 to 10 mice per group.

**Figure 3:**
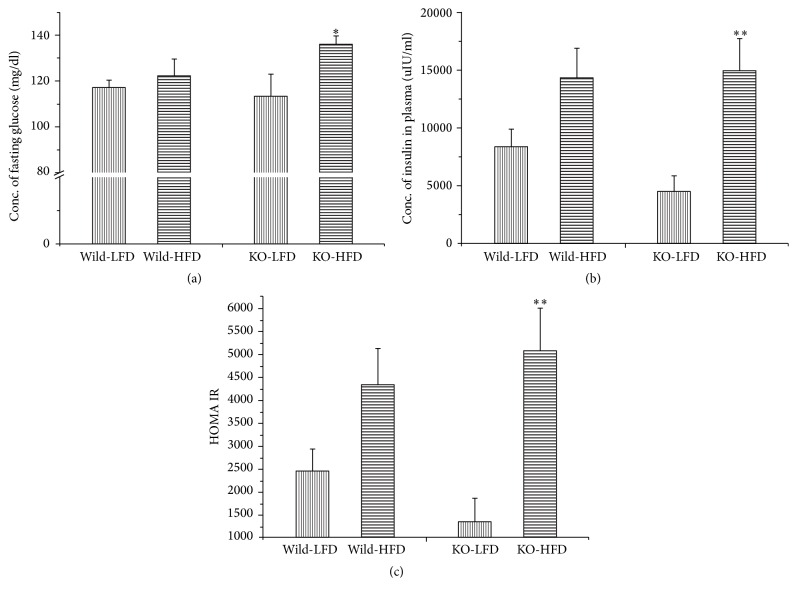
Insulin resistance index in the Mig-6^*f/f*^ and Mig-6^*d/d*^ mice. (a) Fasting plasma glucose concentration. (b) Fasting plasma insulin concentration. (c) HOMA-IR index. The fasting plasma concentration of glucose and insulin were measured at 25 weeks of age. *∗* and *∗∗* indicate *P* < 0.05 and *P* < 0.01 versus LFD treated group in the same genotype, respectively.

The effects of the genotypes and diets on the glucose intolerance and insulin resistance index were measured. In oral glucose tolerance test, the Mig-6^*d/d*^ mice had improved glucose intolerance compared to Mig-6^*f/f*^ mice in both NC- and HFD-fed group, in which the Mig-6^*d/d*^ mice revealed the lower plasma glucose concentrations in all time point and lower area under the curve (AUC) of glucose (*P* < 0.05) (Figure 2(a)). In insulin tolerance tests, Mig-6^*d/d*^ mice had also shown increased glucose clearance rate (*P* < 0.01), indicating the improved insulin resistance, in both NC- and HFD-fed groups (Figure 2(b)). And the HFD contributed to glucose intolerance and insulin resistance in both Mig-6^*f/f*^ and Mig-6^*d/d*^ mice. But the Mig-6^*d/d*^ mice had increased fasting plasma glucose (Figure 3(a)) while the decreased concentration of fasting insulin (Figure 3(b)) and an improved HOMA-IR index (Figure 3(c)) still showed the improved insulin resistance (*P* < 0.01).

### 3.4. Weight of Liver and Fat Tissues

The weights of the liver and fat tissues, obtained at 25 weeks of age, were measured. There were no differences in weight of livers between the genotypes and the ratios of liver to bodyweight of the two genotypes were not significantly different in NC-fed group. But Mig-6^*d/d*^ mice had increased ratios of liver to bodyweight in HFD-fed group, suggesting that HFD had more potential effects in Mig-6^*d/d*^ mice on the liver weight (Table 2).

In the NC-fed animals, the Mig-6^*d/d*^ mice had less subcutaneous fat (*P* < 0.05), visceral fat (*P* < 0.001), and adiposity index (*P* < 0.01) than the Mig-6^*f/f*^ mice. But there were no differences between the genotypes in the HFD-fed group. The HFD significantly increased the fat weight and adiposity index in both genotypes (Table 2). These results indicate that the decreased fat weight leads to lower bodyweight in Mig-6^*d/d*^ mice.

### 3.5. Histological Analyses of Liver and Fats

**Figure 4:**
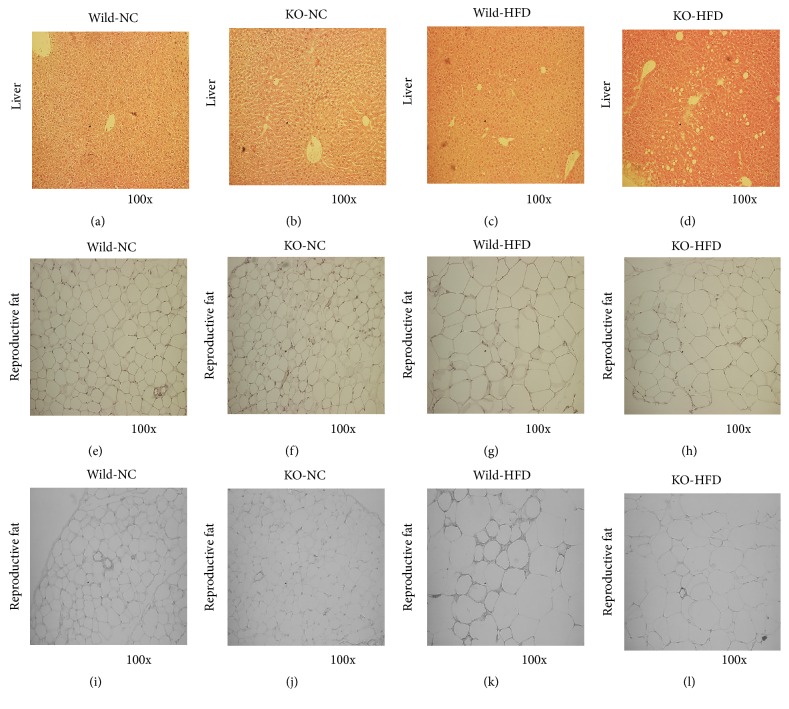
Histological analysis of liver and fat tissues in Mig-6^*f/f*^ and Mig-6^*d/d*^ mice. Microscopic appearance of liver and reproductive fat with H&E staining (×200). (a–d) liver and (e–h) reproductive fat. (i–k) Macrophage-specific staining in the reproductive fat tissues. The IHC of paraffin sections of the reproductive fat tissues was performed using antibody against macrophages (×200).

There were no differences in weight of livers between the genotypes, but H&E staining of liver showed increased vacuoles in the Mig-6^*d/d*^ mice compared to the Mig-6^*f/f*^ (Figures 4(a), 4(b), 4(c) and 4(d)), suggesting that the Mig-6^*d/d*^ mice still have a hepatic steatosis. These results coincided with the increased ratio of liver/bodyweight.

In H&E staining of reproductive fat, the Mig-6^*d/d*^ mice revealed smaller adipocyte volume than Mig-6^*f/f*^, which is consistent with lower fat weight in the Mig-6^*d/d*^ mice (Figures 4(e) and 4(f)). There were no differences in HFD-fed groups (Figures 4(g) and 4(h)). When the reproductive fat tissues were immunostained with anti-macrophage antibody for evaluating the inflammation as an indicator of insulin resistance, there were no differences in NC-fed group (Figures 4(i) and 4(j)) but Mig-6^*d/d*^ mice revealed the lower macrophage deposition in HFD-fed group (Figures 4(k) and 4(l)). The differences were not observed in H&E staining of brown adipose tissue (BAT) between wild and Mig-6^*d/d*^ mice (data not shown).

### 3.6. Western Blot Analyses of Proteins Related to Insulin Resistance

**Figure 5:**
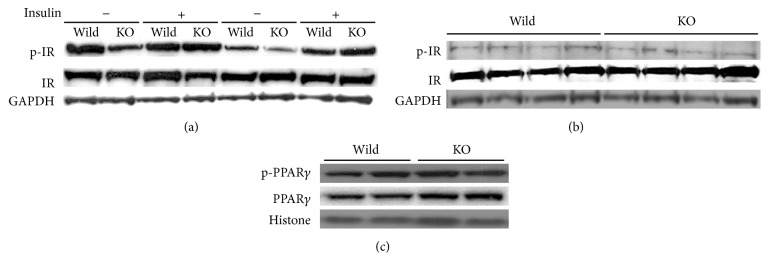
The activation of insulin signaling pathways and PPAR*γ* phosphorylation in Mig-6^*f/f*^ and Mig-6^*d/d*^ mice. The activities of insulin signaling were measured by western blotting using anti-p-IR, anti-IR, and anti-GAPDH antibodies. (a) In the liver (fasting or insulin injection after fasting condition). (b) In the reproductive fat (fasting condition). (c) PPAR*γ* phosphorylation measured by western blotting using anti-p-PPAR*γ*, anti-PPAR*γ*, and anti-histone antibodies in reproductive fat.

The activities of insulin signaling pathway were measured by phosphorylation level of insulin receptor as a parameter of insulin resistance. In liver of fasting condition, p-IR was decreased in Mig-6^*d/d*^ mice, which is consistent with fasting hyperglycemia. But after the mice were intraperitoneally injected with insulin (0.54 IU/kg), the differences in the p-IR levels were not observed between Mig-6^*f/f*^ and Mig-6^*d/d*^ mice (Figure 5(a)). In reproductive fat of fasting condition, no significant differences in p-IR levels were observed between Mig-6^*f/f*^ and Mig-6^*d/d*^ mice (Figure 5(b)). Another insulin resistance index was measured by levels of PPAR*γ* and phospho-PPAR*γ*, an important transcription factor improving the insulin resistance in the fat tissues. The p-PPAR*γ*/PPAR*γ* ratio was not different between the Mig-6^*f/f*^ and Mig-6^*d/d*^ mice (Figure 5(c)).

**Figure 6:**
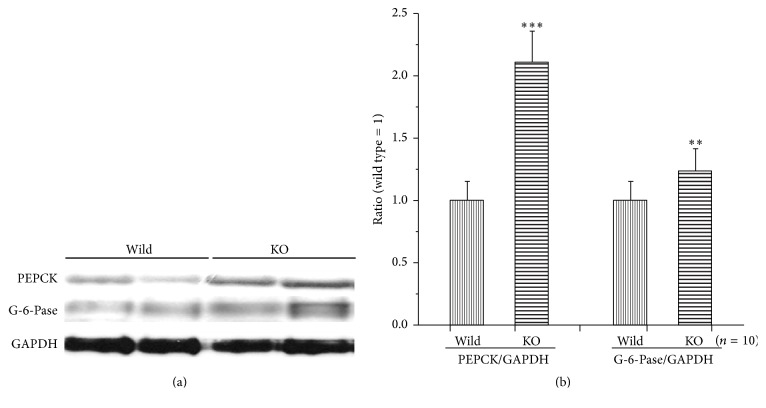
The levels of PEPCK and G6Pase in the liver of Mig-6^*f/f*^ and Mig-6^*d/d*^ mice. (a) Western blotting analysis with anti-PEPCK, anti-G6Pase, and anti-GAPDH antibodies in liver. *∗∗* and *∗∗∗* indicate *P* < 0.01 and *P* < 0.001, versus wild mice, respectively. Data are presented as mean ± SE from 6 to 10 mice per group.

The expression of PEPCK and G-6-Pase, which are rate limiting enzymes of gluconeogenesis in fasting state, was measured. PEPCK and G-6-Pase were increased in the Mig-6^*d/d*^ mice compared to Mig-6^*f/f*^ mice, consisting with elevated fasting glucose concentration (Figure 6).

## 4. Discussion

Mig-6 is highly expressed in liver more than other organs such as fat, muscle, adrenal gland, and lung [1]. According to Ku et al., liver-specific knockout mice of Mig-6 revealed hepatomegaly and hypercholesterolemia, suggesting that EGFR signaling pathway or Mig-6 protein in the liver might play an important role in metabolism of lipids [16]. In this paper, the diabetes-related biomarkers such as plasma concentration of glucose and several lipids and insulin resistance index were analyzed in the wild (Mig-6^*f/f*^) and KO (Mig-6^*d/d*^) mice at 25 weeks of age. As the results of studies, the Mig-6^*d/d*^ mice were characterized as animals with lower bodyweight, higher fasting plasma cholesterol and glucose than Mig-6^*f/f*^ mice, and improved whole-body insulin sensitivity. The Mig-6^*d/d*^ mice have the opposite characteristics in terms of diabetes mellitus and fasting hyperglycemia but improved insulin resistance.

In fasting condition, the blood glucose concentration was increased in the Mig-6^*d/d*^ mice but not significant (Figure 3). These results were not consistent with the previous data from Yoo et al. [17], in which fasting plasma glucose concentration was significantly increased in 8-week-old Mig-6^*d/d*^ mice. The activities of insulin signaling pathway apparently decreased in the liver (Figure 5(a)), accompanied by increased PEPCK proteins (Figure 6) in 25-week-old mice. In addition, the lipid droplets were increased in the liver (Figure 4). These results indicate that insulin resistance might be developed in the liver of Mig-6^*d/d*^ mice, and the activated gluconeogenesis by PEPCK, a rate limiting enzyme in the gluconeogenic pathway [18], might result in inducing hepaitc steatosis. Gluconeogenesis produces glucose from noncarbohydrate carbon substrates such as pyruvate, lactate, glycerol, and glucogenic amino acids for supplying glucose for the brain in the starvation state and is the main pathway controlling the fasting glucose concentration [19, 20] and fasting hyperglycemia is proportional to increased gluconeogenesis [21]. It was reported that PEPCK and SREBP-1c transgenic mice had severe hepatic steatosis, indicating histological steatosis but not steatohepatitis or dyslipidemia in the liver [22, 23]. SREBP-1 (sterol regulatory element-binding protein-1) activating lipid synthesis in the liver was increased in PEPCK transgenic mice [22]. The most of model animals of NAFLD also have fasting hyperglycemia [24]. These reports suggested that the weak fasting hyperglycemia in the 25-week-old Mig-6^*d/d*^ mice seemed to be related to the weaker hepatomegaly than that of the report of Ku et al. [16].

Before this experiment, we expected that the Mig-6^*d/d*^ mice should have impaired glucose homeostasis because the Mig-6^*d/d*^ mice were reported to have hepatomegaly [16] and there were many reports that inhibitors of EGFR signaling pathway, gefitinib, improved the glucose intolerance or insulin resistance in human and experimental animal. But the OGTT and ITT clearly showed that whole-body glucose homeostasis was improved in the Mig-6^*d/d*^ mice (Figure 2) in spite of fatty liver. Therefore, the causes of the improved insulin resistance were analyzed. There was no significant difference in insulin-induced insulin signaling pathway in the liver (Figure 5(a)). Thus, we turn our attention to insulin resistance in fat tissues which is important factor in whole-body insulin resistance. The insulin resistance appears when obesity is developed through chronic intake of high-fat diet. The fat accumulation in tissues and increased inflammation take place at the same time. The inflammation leads to deposition of macrophage in adipose tissue. Macrophage secretes various cytokine and it leads to insulin resistance through decreasing the phosphorylation of IR and PPAR*γ* [25, 26]. We have not detected any difference in insulin signaling pathway in adipose tissue. But macrophage staining was reduced in the Mig-6^*d/d*^ mice compared to wild mice (Figures 4(k) and 4(l)) and PPAR*γ* protein levels were increased in Mig-6^*d/d*^ mice (Figure 6), indicating the improved insulin resistance in fat tissues. The liver and fats are main tissues in development of insulin resistance. The fat contents in the Mig-6^*d/d*^ mice were increased in liver but decreased in fat tissues, accompanied by improved whole-body insulin resistance. This suggests that fat tissues might be more critical in whole-body insulin resistance than the liver.

Additionally, the effects of high-fat diet were revealed to be more significant in the Mig-6^*d/d*^ mice, especially in plasma cholesterol profiles in spite of improved insulin resistance (Table 1), suggesting an important role EGFR signaling pathway in the liver lipid metabolism and insulin resistance. EGFR activation in the Mig-6 deletion mice downregulated CYP7A1 protein expression resulting in hypercholesterolemia [16]. When gefitinib, an EGFR tyrosine kinase inhibitor, was treated in high-fat diet fed Mig-6^*d/d*^ mice, fasting insulin concentration, insulin resistance, and hypercholesterolemia were ameliorated [27]. There was another report that Mig-6 expression was induced by insulin suggesting that insulin signaling might affect EGFR signaling pathway via mig-6 [28]. Those reports suppose that mig-6 may be critical mediator of cross talk between EGFR and insulin signaling pathway.

In summary, mice Mig-6 ablation in the liver result in multiple metabolic phenotypes such as fatty liver, fasting hyperglycemia, and hypercholesterolemia but in lower bodyweight and improved insulin sensitivity. In spite of fatty liver, total fat weight was decreased in the aged Mig-6^*d/d*^ mice. These results suggest that Mig-6 or EGFR signaling pathway should play an important role in the homeostasis of lipid metabolism. Defining the molecular mechanisms by which Mig-6 regulates metabolic syndrome will provide new insight into the development of more effective ways for the treatment and prevention of diabetes and hypercholesterolemia.

## Figures and Tables

**Table 1:** Concentration of plasma biochemical parameters in Mig-6^*f/f*^ and Mig-6^*d/d*^ mice and the effects of high fat diet.

	NC		HFD	
	Mig-6^*f/f*^	Mig-6^*d/d*^	Mig-6^*f/f*^	Mig-6^*d/d*^
Total-cholesterol (mg/dL)	120.0 ± 7.0	170.1 ± 18.4^*∗*^	107.2 ± 11.0	319.0 ± 25.7^*∗∗∗*###^
HDL-cholesterol (mg/dL)	86.5 ± 3.1	133.8 ± 13.7^*∗∗*^	77.4 ± 6.9	244.6 ± 19.4^*∗∗∗*###^
LDL-cholesterol (mg/dL)	11.1 ± 1.7	13.6 ± 2.0	10.9 ± 1.4	35.1 ± 3.5^*∗∗∗*###^
TG (mg/dL)	54.7 ± 7.7	60.2 ± 9.0	57.6 ± 7.0	123.3 ± 17.3^*∗∗*##^
FFA (uEq/dL)	34.2 ± 2.3	34.0 ± 2.3	21.1 ± 1.5^###^	32.3 ± 2.5^##^

*∗*, *∗∗*, and *∗∗∗* indicate *P* < 0.05, *P* < 0.01, and *P* < 0.001, versus Mig-6^*f/f*^, respectively. ## and ### indicate *P* < 0.01 and *P* < 0.001, versus NC of each genotype, respectively. Data are presented as mean ± SE from 9 to 10 mice per group.

**Table 2:** The organ weights of Mig-6^*f/f*^and Mig-6^*d/d*^ mice.

	NC		HFD	
	Mig-6^*f/f*^	Mig-6^*d/d*^	Mig-6^*f/f*^	Mig-6^*d/d*^
Liver	1.11 ± 0.03	1.07 ± 0.06	1.07 ± 0.04	1.23 ± 0.07
Liver ratio (%)	3.65 ± 0.11	4.00 ± 0.23	3.09 ± 0.11^##^	3.85 ± 0.23^*∗∗*^
Subcutaneous fat	0.95 ± 0.08	0.62 ± 0.10^*∗*^	1.67 ± 0.20^##^	1.79 ± 0.22^*∗∗∗*^
Visceral fat	1.36 ± 0.11	0.69 ± 0.11^*∗∗∗*^	2.47 ± 0.22^###^	1.85 ± 0.17^*∗*###^
Adiposity index (%)	8.1 ± 0.51	5.0 ± 0.79^*∗∗*^	13.4 ± 1.16^###^	12.7 ± 1.15^###^

*∗*, *∗∗*, and *∗∗∗* indicate *P* < 0.05, *P* < 0.01, and *P* < 0.001, versus Mig-6^*f/f*^, respectively. ## and ### indicate *P* < 0.01 and *P* < 0.001, versus NC of each genotype, respectively. Data are presented as mean ± SE from 9 to 10 mice per group.
